# A first insight into the occurrence of *Leptospira, Brucella* and *Coxiella burnetii* infections in wild mammals rescued from illegal trade in Ecuador: A proxy for one health conservation policies

**DOI:** 10.1016/j.onehlt.2025.101045

**Published:** 2025-04-18

**Authors:** Ariana Rivera, Marlon Steven Zambrano-Mila, Solon Alberto Orlando, Fabiola Jiménez Valenzuela, Ericka Sanchez, Joselyn Calderon, Manuel González, Angel Sebastian Rodriguez-Pazmiño, Alfonso Marzal, Eliana Molineros, Miguel Angel Garcia-Bereguiain

**Affiliations:** a Instituto Nacional de Salud Pública e Investigación, Guayaquil, Ecuador; b Universidad Yachay Tech, Urcuquí, Ecuador; c One Health Research Group, Universidad de Las Américas, Quito, 170516, Ecuador; d Universidad Espíritu Santo, Guayaquil, Ecuador; e Universidad Católica Santiago de Guayaquil, Guayaquil, Ecuador; f Universidad Ecotec, Guayaquil, Ecuador; g Universidad de Extremadura, Badajoz, Spain; h Widlife Research Group, Universidad Nacional de San Martín, Tarapoto, Peru; i Fundación Proyecto Sacha, Guayaquil, Ecuador; j Ministerio del Ambiente, Agua y Transición Ecológica, Guayaquil, Ecuador

**Keywords:** Leptospirosis, Brucellosis, Ecuador, ELISA, MAT, One health, Trafficking, Q-fever, Wildlife

## Abstract

Illegal wildlife trade is a major concern not only for conservation and animal welfare but also for public health. Human interaction with wild animals increases the risk of zoonotic disease transmission, while exposure of wildlife to humans and domestic animals poses additional threats to biodiversity through the spread of infectious diseases. This issue is particularly critical in tropical, biodiversity-rich countries like Ecuador, often low- and middle-income nations where resources for conservation are limited. In the absence of official data on illegal wildlife trafficking in Ecuador, several non-profit organizations work in collaboration with the Ministry of the Environment to combat this trade and rehabilitate rescued animals, with the ultimate goal of returning them to their natural habitats. In this study, we addressed for the first time in Ecuador the occurrence of three endemic zoonotic pathogens that cause reproductive problems (*Leptospira* spp.*, Brucella* spp.*,* and *Coxiella burnetii*) in wild mammals. A total number of 28 individuals from 15 different species, including primates and felines, were included. All the animals tested positive for antibodies against *Leptospira* spp., and a great diversity of antibodies against pathogenic serovars was found. Moreover, 7.4 % CI: (2.0 %–22.6 %) and 3.7 % CI: (0.6 %–17.7 %) of the animals tested were positive for antibodies against *C. burnetii* and *Brucella* spp., respectively. Our results show that wild mammals are a reservoir for leptospirosis in Ecuador. Also, there is a risk of transmission of *C. burnetii* and *Brucella* spp. from domestic animals to wildlife and vice versa, associated with anthropogenic activities like farming, as those pathogens have a high prevalence in cattle and dogs in Ecuador. In conclusion, wildlife illegal traffic represents a threat to conservation, animal welfare, and public health issues that need to be managed with One Health-inspired policies, like educational programs warning about the risk of wildlife possession for humans and domestic animals.

## 1 Introduction

Leptospirosis, brucellosis, and Q fever are globally distributed bacterial zoonoses that can infect a wide range of vertebrate species. These diseases are commonly associated with reproductive disorders such as abortion and stillbirth, resulting in significant economic losses in livestock production and posing threats to wildlife conservation [[1], [2], [3], [4], [5], [6], [7], [8], [9], [10], [11], [12], [13]]. The pathogenic bacteria responsible for these infections can be transmitted to humans through direct contact with the tissues or bodily fluids of infected animals, as well as through the consumption of raw animal products or contaminated water sources [[1], [2], [3],[10], [11], [12], [13]]. Given their socioeconomic impact and public health relevance, these diseases are subject to mandatory reporting to animal health regulatory authorities.

Leptospirosis is a widely distributed zoonotic infection caused by bacteria of the genus *Leptospira*. Rodents are considered the main reservoir for transmission of the disease to humans, although cattle and dogs have also been identified as important reservoirs in rural settings [[1], [2], [3], [4], [5], [6], [7], [8], [9]]. Transmission among animals or to humans occurs through contaminated soil or water, or via direct contact with fluids such as urine and blood from infected animals [[1], [2], [3]]. Leptospirosis is classified as a neglected tropical disease and is endemic to Ecuador, where high prevalence of antibodies against *Leptospira* spp. has been reported in domestic animals, particularly during seasonal outbreaks associated with the rainy season [10,[14], [15], [16], [17], [18]]. The *Leptospira* genus includes multiple species, serogroups, and serovars that have been traditionally classified as pathogenic, intermediate, and saprophytic according to their virulence [[1], [2], [3], [4], [5], [6], [7], [8], [9]]. The prevalence of *Leptospira* spp. infection is still addressed through serological testing using the microscopic agglutination test (MAT), the gold standard method [18].

Brucellosis is another neglected zoonosis prevalent worldwide, including South American countries [[10], [11], [12], [13]]. Bacteria from the *Brucella* genus cause brucellosis, with several species including *B. abortus, B. melitensis, and B. suis* that can infect animals and humans [[11], [12], [13]]. The most common routes of infection for brucellosis in humans and animals are through aerosol inhalation and contact with secretions from infected animals [[11], [12], [13],^19^]. Although this infection rarely occurs in humans in high-income countries, brucellosis is still a public health issue in low- and middle-income countries where diagnosis is insufficient [10,12,[19], [20], [21]]. Although there is a national program for animal brucellosis surveillance in Ecuador, there are reports showing that bovine brucellosis is endemic and antibodies against *Brucella* spp. are highly prevalent with values up to 17 % in cattle [12,[19], [20], [21]].

Another zoonotic disease that has gained attention recently is Q fever, having an impact on a wide range of animals. It is also associated with economic losses in dairy farming [[11], [12], [13]]. The route of transmission is through contaminated dust, soil, or direct contact with secretions from infected animals with the bacteria *Coxiella burnetii* [[11], [12], [13],^21^]. While much of the research on Q fever has focused on high-income countries, data from low- and middle-income countries are still scarce [[11], [12], [13]]. In Ecuador, there are a few pioneer reports showing a high prevalence of antibodies against *C. burnetii* in cattle and suggesting potential underdiagnoses in humans, particularly among those exposed occupationally within the farming industry [12,13,21].

As described above, leptospirosis, Q fever, and brucellosis are endemic and neglected zoonotic diseases in Ecuador [10]. Although several reports have highlighted their impact on livestock production and public health, their effects on wild fauna remain largely unstudied. Ecuador is one of the world's most biodiverse countries, home to numerous iconic and endangered species, including several primates [17,21]. Illegal wildlife trade poses a significant threat to conservation efforts. Despite recognition of the issue by conservation authorities and non-profit organizations, there is a lack of official data on the number of animals held in captivity as a result of illegal trade.

This activity not only threatens biodiversity through direct exploitation but also increases the risk of pathogen transmission from humans and domestic animals to wildlife, particularly diseases that affect reproductive success. The danger is twofold: exposure of wildlife to new pathogens, and the potential spillover of infections from wildlife back to humans and domestic animals. Furthermore, reintroducing animals rescued from illegal trade into the wild may inadvertently facilitate the spread of pathogens to new areas, potentially leading to outbreaks and the emergence of diseases that could impact native wildlife, livestock, and human populations [[22], [23], [24]].

These risks must be carefully evaluated before any reintroduction efforts. Overall, the complex interactions between humans, domestic animals, and wildlife—driven by environmental degradation and illegal trade—pose significant threats to public health, animal production, and biodiversity. Addressing these challenges requires integrated surveillance and control strategies under a One Health approach [17,18,21].

Given this scenario, this study aimed to investigate, for the first time in Ecuador, the presence of antibodies against *Leptospira* spp., *Brucella* spp., and *Coxiella burnetii* in wild mammals housed in wildlife rescue centers.

## 2 Materials and methods

### 2.1 Study setting and sample collection

This study was conducted at the rescue and conservation center “Mansión Mascota–Fundación Proyecto Sacha,” located in the city of Guayaquil, in southwestern Ecuador. The center collaborates with the Ministry of the Environment of Ecuador to receive wildlife rescued from illegal trafficking or found injured, providing veterinary care and rehabilitation with the goal of eventual reintroduction into their natural habitats when possible. For this study, sample collection took place in 2019. A total of 28 wild mammals were included, representing various species of primates, felines, and other taxa (Table 1). Blood samples were collected by the center's veterinary staff under anesthesia. Species identification was performed based on morphological characteristics. Blood samples were centrifuged at 5000 rpm for 5 min to obtain serum for further analysis.

**Table 1:**
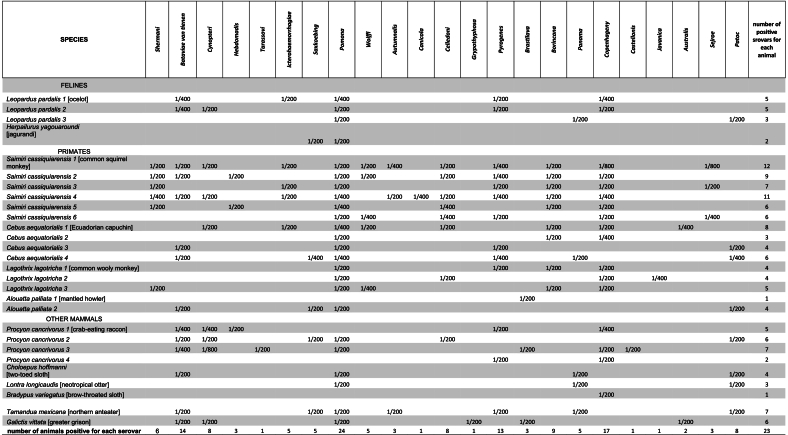
MAT results for the 28 wild mammals included in this study. Only the 23 *Leptospira* serovars that were positive for any of the samples tested are included. Number refers to the titer dilution in MAT.

### 2.2 Microscopic agglutination test for the detection of anti-Leptospira antibodies

The Microscopic Agglutination Test (MAT) was conducted using a panel of 24 *Leptospira* strains representing various serogroups and serovars, as detailed in Supplementary Table 1. The test was performed at the *Laboratorio Nacional de Referencia para Zoonosis* of the *Instituto Nacional de Salud Pública e Investigación* in Guayaquil. This laboratory specializes in the analysis of human samples using MAT and employs a strain panel established in accordance with Pan American Health Organization (PAHO) guidelines [17,25].

For serum screening, an initial dilution of 1:200 was used. Samples testing positive at this dilution were subsequently evaluated using serial dilutions ranging from 1:200 to 1:3200. The highest dilution showing ≥50 % agglutination under 40× magnification was recorded as the endpoint titer. A serum sample was considered reactive if it demonstrated at least 50 % agglutination at any of the tested dilutions.

### 2.3 Q-fever and brucellosis serology

Commercial indirect ELISA kits ID Screen® Brucellosis Serum Indirect and ID Screen® Q Fever Indirect Multi-species (IDVet, Montpellier, France) were used to detect antibodies against *Brucella* spp. (*B. abortus*, *B. melitensis*, and *B. suis*) and *Coxiella burnetii*, respectively. The reported sensitivity and specificity of the Brucella ELISA were 100 % (95 % CI: 89.57–100 %) and 99.74 % (95 % CI: 99.24–99.91 %), respectively, while for the Q fever ELISA, both sensitivity and specificity were 100 % (95 % CI: 89.28–100 % and 97.75–100 %, respectively).

The Brucella ELISA has been validated for use in cattle (*B. abortus*), sheep and goats (*B. melitensis*), and pigs (*B. suis*). The Q fever ELISA has been validated for detecting antibodies against *C. burnetii* in sheep, goats, and cattle. However, according to the manufacturer, both kits use a conjugate that recognizes anti-mammalian antibodies, enabling potential cross-species detection in other mammalian wildlife species.

### 2.4 Statistical analysis

Given the small sample size in our study, the Wilson Score Interval was used instead of the normal approximation, which assumes a large sample size. The Wilson method offers more reliable accuracy, particularly when dealing with extreme proportions, such as values close to 0 % or 100 %.

### 2.5 Ethics statements

This study was conducted following Ecuadorian regulations for disease diagnosis and surveillance in wildlife. “Fundación Mascota–Proyecto Sacha” held official permits for wildlife management and sample collection for disease diagnostics, granted by the *“Ministerio del Ambiente, Agua y Transición Ecológica”* of Ecuador. In addition, the implementation of the MAT analysis at the “Laboratorio Nacional de Referencia para Zoonosis” was approved by the “Instituto Nacional de Salud Pública e Investigación” (INSPI) of Ecuador.

## 3 Results

### 3.1 Seroprevalence of anti-Leptospira spp. antibodies and diversity of serovars

All 28 wild mammals included in this study tested positive for *Leptospira* spp. by the MAT assay, with titers ranging from 1:200 to 1:800 across 23 different serovars (Table 1). Every sample showed reactivity to at least two serovars, and 25 animals tested positive for three or more (Table 2). Notably, the squirrel monkey (*Saimiri cassiquensis*) exhibited seropositivity to 12 different serovars (Table 2).

**Table 2:** Distribution of animals (28) according to the number of positive *Leptospira* serovars (sv mean serovar).

	Number of animals (%)	95 % CI
1sv	2 (7 %)	(1.92 %, 22.46 %)
2sv	2 (7 %)	(1.92 %, 22.46 %)
3sv	3 (10.7 %)	(3.70 %, 27.18 %)
4sv	5 (17.8 %)	(7.84 %, 35.53 %)
5sv	4 (14.2 %)	(5.65 %, 31.39 %)
6sv	5 (17.8 %)	(7.84 %, 35.53 %)
7sv	3 (10.7 %)	(3.70 %, 27.18 %)
8sv	1 (3.6 %)	(0.64 %, 17.75 %)
9sv	1 (3.6 %)	(0.64 %, 17.75 %)
11sv	1 (3.6 %)	(0.64 %, 17.75 %)
12sv	1 (3.6 %)	(0.64 %, 17.75 %)

Among the *Leptospira* serovars included in the MAT panel, Copenhageni, Sejroe, and Cynopteri showed the highest titers, reaching up to 1:800 in samples from raccoons (*Procyon cancrivorus*) and squirrel monkeys (Table 3). The most prevalent serovars detected were Pomona (80 %; 95 % CI: 68.51 %–94.30 %), Copenhageni (56.7 %; 95 % CI: 42.41 %–76.43 %), Bataviae (46.7 %; 95 % CI: 32.63 %–67.37 %), and Pyrogenes (43.3 %; 95 % CI: 29.53 %–64.19 %) (Table 3).

**Table 3:** Distribution *of Leptospira* serovars and titer ranges for MAT for the 28 wild mammals included in the study.

N°	Serogroup	Serovar	Number of animals (%) IC (95 %)	Titer range
1.	**Pomona**	**Pomona**	**24(80 %)** **(68.51 %–94.30 %)**	**1/200–1/400**
2.	**Icterohaemorrhagiae**	**Copenhageni**	**17(56.7 %)** **(42.41 % – 76.43 %)**	**1/200–1/800**
3.	**Bataviae**	**Bataviae**	**14(46.7 %)** **(32.63 % – 67.37 %)**	**1/200–1/400**
4.	**Pyrogenes**	**Pyrogenes**	**13(43.3 %)** **(29.53 % – 64.19 %)**	**1/200–1/400**
5.	Hebdomadis	Borincana	9(30 %) (17.93 % – 50.66 %)	1/200
6.	Cynopteri	Cynopteri	8(26.7 %) (15.25 % – 47.06 %)	1/200–1/800
7.	Celledoni	Celledoni	8 (26.7 %) (15.25 % – 47.06 %)	1/200–1/400
8.	Semaranga	Patoc	8(26.8 %) (15.25 % – 47.06 %)	1/200–1/400
9.	Shermani	Shermani	6(20 %) (10.21 % – 39.54 %)	1/200
10.	Icterohaemorrhagiae	Icterohaemorrhagiae	5(16.3 %) (7.88 % – 35.59 %)	1/200
11.	Sejroe	Saxkoebing	5(16.7 %) (7.88 % – 35.59 %)	1/200–1/400
12.	Sejroe	Wolffi	5(16.7 %) (7.88 % – 35.59 %)	1/200–1/400
13.	Panama	Panama	5(16.7 %) (7.88 % – 35.59 %)	1/200
14.	Australis	Bratislava	3(10 %) (3.71 % – 27.20 %)	1/200
15.	Hebdomadis	Hebdomadis	3(10 %) (3.71 % – 27.20 %)	1/200
16.	Autumnalis	Autumnalis	3(10 %) (3.71 % – 27.20 %)	1/200–1/400
17.	Sejroe	Sejroe	3(10 %) (3.71 % – 27.20 %)	1/200–1/800
18.	Australis	Australis	2(6.7 %) (1.98 % – 22.65 %)	1/200–1/400
19.	Tasassovi	Tasassovi	1(3.3 %) (0.63 % – 17.71 %)	1/200
20.	Canicola	Canicola	1(3.3 %) (0.63 % – 17.71 %)	1/1400
21.	Grippotyphosa	Grippotyphosa	1 (3.3 %) (0.63 % – 17.71 %)	1/200
22.	Javanica	Javanica	1 (3.3 %) (0.63 % – 17.71 %)	1/400
23.	Ballum	Castellonis	1(3.3 %) (0.63 % – 17.71 %)	1/200

### 3.2 Seroprevalence of anti-Brucella spp. and *Coxiella burnetii* antibodies

Only one sample, from a male anteater (*Tamandua mexicana*), tested positive for antibodies against *Brucella* spp. by ELISA, representing a seroprevalence of 3.57 % (95 % CI: 0.63 %–17.71 %). This individual had a history of prolonged confinement and close contact with domestic farm animals before it arrived at the rescue center. Notably, the same anteater also tested positive for antibodies against *C. burnetii*.

Additionally, a male ocelot (*Leopardus pardalis*) tested positive for *C. burnetii* antibodies by ELISA. The overall seroprevalence of exposure to *C. burnetii* in our study population was 7.4 % (95 % CI: 1.98 %–22.6 %). This ocelot had no evidence of prior captivity and was brought to the rescue center after being injured on a highway.

## 4 Discussion

Our study revealed a high seroprevalence of antibodies against a diverse range of pathogenic *Leptospira* species among 28 wild mammals housed at a rescue center in Guayaquil, Ecuador. In addition, some individuals also tested positive for antibodies against *Brucella* spp. and *C. burnetii*. Notably, one anteater, previously kept in prolonged confinement as a pet and in close contact with domestic animals, tested positive for both *Brucella* spp. and *C. burnetii*. In contrast, the ocelot that tested positive for *C. burnetii* had been admitted to the center after sustaining injuries from a vehicle collision. Aside from trauma related to the accident, the animal showed no signs of prior captivity, suggesting it had been living in the wild.

Regarding leptospirosis, our findings support previous reports confirming the endemic nature of this disease in Ecuador, with multiple animal reservoirs including stray dogs, cats, rats, livestock, and captive wild mammals [[14], [15], [16], [17]]. Recent outbreaks affecting humans in the Coastal Region have further highlighted its public health relevance, with stray dogs and rats identified as key reservoirs. These events underscore the urgent need for an integrated One Health surveillance approach to reduce the burden of leptospirosis in humans and improve animal health outcomes [18].

The results of this study also align with our earlier report describing leptospirosis in captive wild fauna in Ecuador [17]. In that study, we examined a small group of wild animals housed in a mixed-use facility where they shared space with domestic animals, with no biosecurity measures or veterinary oversight. In contrast, the current study involved a larger and more diverse cohort of wild mammals, including individuals rescued from illegal wildlife trade and others injured in vehicle collisions. All animals in this study received veterinary care upon admission to the rescue center, where quarantine and isolation protocols were properly implemented. Samples for zoonotic disease surveillance were collected immediately upon arrival.

In this context, our findings suggest that the high prevalence of leptospirosis and the diversity of circulating pathogenic serovars are associated both with wild mammals trafficked illegally and with those originating from natural habitats. Wild mammals in Ecuador therefore represent an important reservoir of leptospirosis, posing a potential transmission risk to humans and domestic animals. This is consistent with other recent reports from Ecuador, which document the presence of diverse *Leptospira* serovars across various animal species, including dogs, horses, cattle, pigs, rats, and wild mammals in captivity [[14], [15], [16], [17], [18],[26], [27], [28], [29], [30]].

Furthermore, a recent meta-analysis comprising 79 studies across Latin America and covering 186 wild species confirmed that leptospirosis is widespread in wildlife throughout all major biomes of the region [31]. Anthropogenic pressures such as farming and habitat degradation expose wild fauna to *Leptospira* through contact with domestic animals. In turn, illegal wildlife trade exposes humans and domestic animals to *Leptospira* strains circulating in wildlife, some of which may not be covered by currently available vaccines. Altogether, leptospirosis represents a paradigmatic case demonstrating the need for a comprehensive One Health approach to safeguard public health, livestock, and wildlife conservation.

The findings related to brucellosis and Q fever are particularly noteworthy. The detection of antibodies against *Brucella* spp. and *C. burnetii* in a captive anteater highlights the potential zoonotic risk associated with illegal wildlife trade. Close contact between trafficked wild animals and humans or domestic species can facilitate the transmission of zoonotic pathogens [17,[19], [20], [21]]. Interestingly, a 2015 report also documented a case of brucellosis in an anteater in Brazil [32]. Furthermore, a recent meta-analysis comprising 68 publications up to 2019 reported high brucellosis prevalence in wild species such as American bison and Alpine ibex, supporting the enzootic potential of brucellosis in wildlife globally [33].

Regarding Q fever, a separate meta-analysis encompassing 113 studies from Latin America up to 2022 revealed that knowledge about *C. burnetii* infections in wildlife remains fragmented. However, the pathogen has already been detected in multiple taxonomic groups, suggesting its potential to become enzootic in wild populations [34].

As described in the introduction, both Q fever and brucellosis are widely distributed among domestic animals in Ecuador [12,13,[19], [20], [21]]. Ecosystem degradation caused by agricultural expansion may increase wildlife exposure to these pathogens [17]. Conversely, the establishment of new wildlife reservoirs could hinder ongoing efforts to control these diseases in livestock.

While wildlife rescue centers primarily aim to rehabilitate and reintroduce animals into their natural habitats, release decisions should not be based solely on an animal's physical recovery or survival fitness. It is also essential to consider the risk of introducing novel pathogens into wild populations. Therefore, illegal wildlife trade and the management of rescued animals must be integrated into national surveillance and control programs for brucellosis and Q fever. As with leptospirosis, an integrative One Health approach is critical to improving the health of humans, livestock, and ecosystems.

Our study has some limitations that should be acknowledged. First, the sample size of 28 animals is too small to draw robust epidemiological conclusions about the impact of these zoonotic diseases on wildlife in Ecuador. Second, all animals were sampled from a single rescue center located in the Coastal Region, which introduces potential geographical bias. Therefore, further research involving a larger number of wild animals from multiple regions across the country is needed to better understand the prevalence and impact of leptospirosis, brucellosis, and Q fever in Ecuador's wildlife. We hope that this descriptive study serves as a foundation for future investigations in this important area.

Despite the limitations noted, our findings allow us to propose several recommendations to help mitigate zoonotic spillovers linked to wildlife trafficking and to strengthen wildlife and ecosystem conservation efforts in Ecuador. First, it is essential to reinforce the enforcement of anti-trafficking laws by providing adequate resources and support to both law enforcement and Ministry of Environment personnel. Second, community-based conservation and education initiatives should be implemented to raise awareness and foster public engagement in wildlife protection. Third, zoonotic surveillance in wildlife and livestock should be integrated with national public health monitoring systems. Ultimately, a coordinated One Health approach is needed (bringing together the Ministry of Health, Ministry of Agriculture, and Ministry of Environment) to develop and sustain a comprehensive national surveillance program for zoonotic diseases.

## CRediT authorship contribution statement

**Ariana Rivera:** Writing – original draft, Resources, Project administration, Methodology, Investigation. **Marlon Steven Zambrano-Mila:** Writing – original draft, Resources, Project administration, Methodology, Investigation. **Solon Alberto Orlando:** Writing – review & editing, Supervision, Methodology, Investigation, Data curation, Conceptualization. **Fabiola Jiménez Valenzuela:** Writing – review & editing, Resources, Project administration, Investigation. **Ericka Sanchez:** Writing – review & editing, Methodology, Investigation. **Joselyn Calderon:** Writing – review & editing, Methodology, Investigation. **Manuel González:** Writing – review & editing, Methodology, Investigation. **Angel Sebastian RodrIguez-Pazmiño:** Writing – review & editing, Methodology, Investigation. **Alfonso Marzal:** Writing – review & editing, Validation, Supervision. **Eliana Molineros:** Writing – review & editing, Project administration, Methodology, Investigation, Conceptualization. **Miguel Angel Garcia-Bereguiain:** Writing – review & editing, Writing – original draft, Resources, Funding acquisition, Formal analysis, Conceptualization.

## Declaration of competing interest

The authors declare that they have no known competing financial interests or personal relationships that could have appeared to influence the work reported in this paper.

## Data Availability

Data will be made available on request.

## Appendix A Supplementary data

Supplementary material Image 1

## References

[1] Adler Ben (2011). Pathogenesis of leptospirosis: the influence of genomics. Vet. Microbiol..

[2] Ellis William A. (2015). Current Topics in Microbiology and Immunology.

[3] Faine S., World Health Organization (1982).

[4] Sales, dos Santos Indiara (2012). *Leptospira* and *Brucella* antibodies in collared anteaters (*Tamandua Tetradactyla*) in Brazilian Zoos. J. Zoo Wildl. Med..

[5] Straub Mary H. (2020). Raccoons (*Procyon Lotor*) and striped skunks (*Mephitis Mephitis*) as potential reservoirs of Leptospira Spp. in California. Vect. Borne Zoonot. Dis..

[6] Tan Ching Giap (2014). Neglected leptospirosis in raccoons (*Procyon Lotor*) in Indiana, USA. Vet. Q..

[7] Ullmann Leila Sabrina (2012). Serologic survey for *Leptospira* spp. in captive neotropical felids in foz do Iguaçu, Paraná, Brazil. J. Zoo Wildl. Med..

[8] Jobbins S.E., Alexander K.A. (2015). Evidence of Leptospira sp. infection among a diversity of African wildlife species: beyond the usual suspects. Trans. R. Soc. Trop. Med. Hyg..

[9] Vieira Anahi S. (2018). A systematic review of leptospirosis on wild animals in Latin America. Trop. Anim. Health Prod..

[10] Gestal Cartelle, Monica (2015). Epidemiology of tropical neglected diseases in Ecuador in the last 20 years. PLoS ONE.

[11] Eldin Carole (2017). From Q fever to *Coxiella Burnetii* infection: a paradigm change. Clin. Microbiol. Rev..

[12] Changoluisa D. (2019). Serology for Neosporosis, Q fever and brucellosis to assess the cause of abortion in two dairy cattle herds in Ecuador. BMC Vet. Res..

[13] Echeverría G. (2019). Serological evidence of Coxiella burnetii infection in cattle and farm workers: is Q fever an underreported zoonotic disease in Ecuador?. Infect. Drug Resist..

[14] Barragan, Veronica, et al. “High leptospira diversity in animals and humans complicates the search for common reservoirs of human disease in rural Ecuador.” PLoS Negl. Trop. Dis., vol. 10, no. 9, 2016, p. e0004990, doi:10.1371/journal.pntd.0004990. PMC5021363 27622673

[15] Id Calvopiña, Manuel (2022). Leptospirosis: morbidity, mortality, and spatial distribution of hospitalized cases in Ecuador. A nationwide study 2000–2020. PLoS Negl. Trop. Dis..

[16] Miller Erin (2021). Leptospira in river and soil in a highly endemic area of Ecuador. BMC Microbiol..

[17] Orlando Solon Alberto (2020). High seroprevalence of anti-Leptospira Spp. antibodies in domestic and wild mammals from a mixed use rescue center in Ecuador: lessons for ‘one health’ based conservation strategies. One Health.

[18] Orlando, Alberto Solon (2024). Leptospirosis outbreak in Ecuador in 2023: a pilot study for surveillance from a one health perspective. One Health.

[19] Carbonero A. (2018). Seroprevalence and risk factors associated with brucella seropositivity in dairy and mixed cattle herds from Ecuador. Trop. Anim. Health Prod..

[20] Poulsen Keith P. (2014). Brucellosis in dairy cattle and goats in Northern Ecuador. Am. J. Trop. Med. Hyg..

[21] Rodriguez-Pazmiño A.S. (2024). A first insight into seropositivity and risk factors for Brucella spp. and *Coxiella burnetii* in free-roaming dogs in Ecuador. One Health.

[22] Bezerra-Santos M.A., Mendoza-Roldan J.A., Thompson R.C.A., Dantas-Torres F., Otranto D. (2021). Illegal wildlife trade: a gateway to zoonotic infectious diseases. Trends Parasitol..

[23] Rush E.R., Dale E., Aguirre A.A. (2021). Illegal wildlife trade and emerging infectious diseases: pervasive impacts to species, ecosystems and human health. Animals.

[24] Marzal A., Magallanes S., Salas-Rengifo T., Muriel J., Navarro C., Vecco D., Guerra-Saldaña C., Mendo L., Paredes V., González-Blázquez M., García-Longoria L., Díez-Fernández A. (2024). Prevalence and diversity of avian malaria parasites in illegally traded white-winged parakeets in Peruvian Amazonas. Anim. Conserv..

[25] (2003). Human Leptospirosis : Guidance for Diagnosis, Surveillance and Control.

[26] Barragan V., Nieto N., Keim P., Pearson T. (2017). Meta-analysis to estimate the load of Leptospira excreted in urine: beyond rats as important sources of transmission in low-income rural communities. BMC Res. Notes.

[27] Burgos Macias D.I., Pérez Ruano M., Bulnes Goicochea C.A., Zambrano Aguayo M.D., Sandoval Valencia H.P., Falconi Flores M.A. (2019). Determination of the seroprevalence of Leptospira spp. and the main serovars circulating in cattle in the province of Manabí. Ecuador. Rev. Off. Int. Epizoot..

[28] Orlando S.A., Paez Martinez K., Sanchez E., de la Cruz C., Calderon J., Arcos F., Torres-Lasso P., Calvopiña M., Garcia-Bereguiain M.A. (2023). Racehorses from a breeding farm in tropical Ecuador have a high seroprevalence of anti-Leptospira spp. antibodies: a paradigm for leptospirosis management from a one health perspective. Front. Trop. Dis..

[29] Chiriboga J., Barragán V., Arroyo G., Sosa A., Birdsell D.N., España K., Mora A., Espín E., Mejía M.E., Al., J.C.E. (2015). High prevalence of intermediate Leptospira spp. DNA in febrile humans from urban and rural Ecuador. Emerg. Infect. Dis..

[30] Ruano M.P., Burgos-Macıas D.I., Goicochea C.A.B., Zambrano Aguayo M.D., Sandoval Valencia H.P., Falconi Flores M.A., Vera Loor L.A., Revelo Ruales A.P., Fonseca-Rodríguez O. (2020). Seroprevalence and risk factors of bovine leptospirosis in the province of Manabí, Ecuador. Comp. Immunol. Microbiol. Infect. Dis..

[31] Vieira A.S., Pinto P.S., Lilenbaum W. (2018). A systematic review of leptospirosis on wild animals in Latin America. Trop. Anim. Health Prod..

[32] Miranda F.R. (2015). Open-access Serosurvey of *Leptospira interrogans, Brucella abortus* and *Chlamydophila abortus* infection in free-ranging giant anteaters (*Myrmecophaga tridactyla*) from Brazil. Pesqui. Vet. Bras..

[33] Dadar M., Shahali Y., Fakhri Y., Godfroid J. (2020). The global epidemiology of Brucella infections in terrestrial wildlife: a meta-analysis. Transbound. Emerg. Dis..

[34] Epelboin L., Mioni De Souza Ribeiro, M., Couesnon, A. (2023). Coxiella burnetii infection in livestock, pets, wildlife, and ticks in Latin America and the Caribbean: a comprehensive review of the literature. Curr. Trop. Med. Rep..

