# Supplementary material for: A first insight into the occurrence of Leptospira, Brucella and Coxiella burnetii infections in wild mammals rescued from illegal trade in Ecuador: A proxy for one health conservation policies
Source: One Health. 2025 Apr 18;20:101045. doi: 10.1016/j.onehlt.2025.101045 (PMC12434589; doi:10.1016/j.onehlt.2025.101045)
Supplement: Supplementary file 1 — Supplementary material [file mmc1.docx]

**Supplementary Table 1. Panel of 24 reference strains for multiple serovars of *Leptospira* used for the MAT in this study.**

| N° | specie | Serogroup | Serovar | strain |
| --- | --- | --- | --- | --- |
|  | **Pathogenic** |  |  |  |
| 1 | *L. santarosai* | Shermani | Shermani | 1342 k |
| 2 | *L. interrogans* | Bataviae | Bataviae | Van tienen |
| 3 | *L. kirschneri* | Cynopteri | Cynopteri | 3522c |
| 4 | *L. interrogans* | Hebdomadis | Hebdomadis | Hebdomadis |
| 5 | *L. borgpetersenii* | Tasassovi | Tasassovi | Perepelitsin |
| 6 | *L. interrogans* | Icterohaemorrhagiae | Icterohaemorrhagiae | Ictero I |
| 7 | *L. borgpetersenii* | Sejroe | Saxkoebing | Mus 24 |
| 8 | *L. interrogans* | Sejroe | Hardjo | Hardjoprajtino |
| 9 | *L. interrogans* | Pomona | Pomona | Pomona |
| 10 | *L. interrogans* | Sejroe | Wolffi | 3705 |
| 11 | *L. interrogans* | Autumnalis | Autumnalis | Akiyami A |
| 12 | *L. interrogans* | Canicola | Canicola | Hond Utrecht IV |
| 13 | *L. weilii* | Celledoni | Celledoni | Celledoni |
| 14 | *L. kirschneri* | Grippotyphosa | Grippotyphosa | Moska V |
| 15 | *L. interrogans* | Pyrogenes | Pyrogenes | Salinem |
| 16 | *L. interrogans* | Australis | Bratislava | Jez Bratislava |
| 17 | *L. santarosai* | Hebdomadis | Borincana | HS 622 |
| 18 | *L. noguchii* | Panama | Panama | CZ 214 |
| 19 | *L. interrogans* | Icterohaemorrhagiae | Copenhageni | M20 |
| 20 | *L. borgpetersenii* | Ballum | Castellonis | Castellon 3 |
| 21 | *L. borgpetersenii* | Javanica | Javanica | Veldrat Batavia 46 |
| 22 | *L. interrogans* | Australis | Australis | Ballico |
| 23 | *L. Borgpetersenii* | Sejroe | Sejroe | M 84 |
| 24 | *L. interrogans* | Djasiman | Djasiman | Djasiman |
